# Complex immunotherapy-mediated response patterns depicted on serial ^18^F-FDG PET imaging of Hodgkin lymphoma patient undergoing pembrolizumab therapy

**DOI:** 10.22038/aojnmb.2025.86889.1621

**Published:** 2025

**Authors:** Mohanad Badarneh, Ahmed Saad Abdlkadir, Mohammad Makoseh, Alaa Abufara, Akram Al-Ibraheem

**Affiliations:** 1 Department of Nuclear Medicine, King Hussein Cancer Center (KHCC), Amman, Jordan; 2 Department of Medicine, King Hussein Cancer Center (KHCC), Amman, Jordan; 3 School of Medicine, The University of Jordan, Amman, Jordan

**Keywords:** PET/CT, ^18^F-FDG, Immunotherapy, Abscopal effect Pseudoprogression

## Abstract

Over the past decade, the role of ^18^F-Fluorodeoxyglucose (^18^F-FDG) positron emission tomography/computed tomography (PET/CT) in assessing treatment response for lymphoma patients undergoing immunotherapy has been extensively investigated. The advent of immunotherapy has challenged the utility of the established Lugano criteria, prompting the development of novel immunotherapy-specific response criteria now employed in clinical practice. Following ^18^F-FDG PET/CT evaluation after up to eight immunotherapy cycles, patients are typically transitioned to conventional imaging modalities if immunotherapy continuation is warranted. This case report illustrates the application of serial ^18^F-FDG PET/CT in monitoring a 67-year-old patient with Hodgkin lymphoma receiving immunotherapy, revealing complex, atypical response patterns unresolved by existing criteria. Notably, two episodes of pseudoprogression occurred at two distinct time points, one during cycle 5 and the other during cycle 22. Furthermore, an immunotherapy-enhanced abscopal effect was seen during radio-immunotherapy, leading to short-term disease remission. Our findings suggest that ^18^F-FDG PET/CT provides superior predictive value in delineating heterogeneous response patterns, thereby informing critical decisions regarding immunotherapy cessation or adjunctive therapeutic interventions.

## Introduction

 The use of immunotherapy in lymphoma is becoming more prevalent over the years. To date, a broad range of immunotherapy agents becomes available in clinical practice subsequent to their approval by the Food and Drug Administration (FDA) (1). Monoclonal antibodies such as brentuximab vedotin (BV) and immune checkpoint inhibitors (ICI) including pembrolizumab, as well as chimeric antigen receptor therapy (CAR-T), are commonly used in Hodgkin lymphoma (HL) patients, mainly in relapsed or refractory cases (2). ^18^F-FDG positron emission tomography coupled with computed tomography (PET/CT) plays a fundamental role in the management of lymphoma patients (3). ^18^F-FDG PET/CT is primarily used for staging, interim evaluation (i-PET/CT), end-of-therapy evaluation (Eot PET/CT), and the assessment of recurrent disease (4). The Lugano Classification, published in 2014, is the standard visual interpretation criteria for EoT and i-PET/CT in ^18^F-FDG-avid lymphomas (4). Response patterns include complete metabolic response (CMR), partial metabolic response (PMR), stable metabolic disease (SMD), and progressive metabolic disease (PMD) (4).

 Utilizing ^18^F-FDG PET/CT in the evaluation of patients receiving immunotherapy poses several challenges, evident by the emergence of new atypical response patterns not explained by the Lugano criteria (3, 4). To address these challenges, the Lymphoma Response to Immunomodulatory Therapy Criteria (LYRIC) and Response Evaluation Criteria in Lymphoma (RECIL) have been introduced (5, 6). If the decision is to continue treatment after confirming the response pattern with ^18^F-FDG PET/CT, patients are usually followed up by serial CT scans every three months (7). 

 However, in unique cases, patients may be followed up by serial ^18^F-FDG PET/CT scans instead (8). Both LYRIC and RECIL criteria have managed to explain some of these response patterns, though certain patterns still remain unexplained by any of the criteria (9).

## Case Report

**Figure 1:**
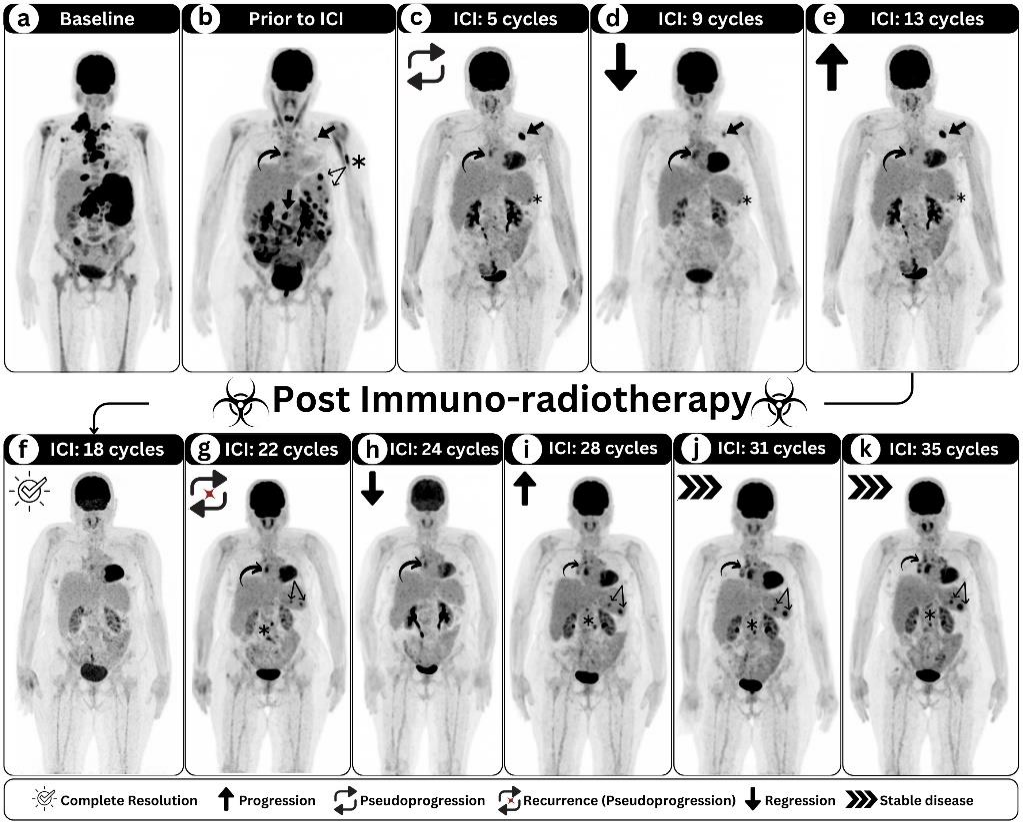
(**a**- **k**) Serial maximum intensity projection images of ^18^F-FDG PET/CT offered prior to immunotherapy initiation up until administration of 35 cycles of pembrolizumab

A 67-year-old female patient first presented with weight loss and night sweats; diagnosed in 2020 with mixed cellularity HL after performing biopsy from cervical lymph node. Staging ^18^F-FDG PET/CT scan showed hypermetabolic lymphomatous disease involving lymph node groups above and below the diaphragm with spleen and bone marrow involvement, indicative of stage IV HL (Figure 1a). Over the past two years, the patient had been on three chemotherapy protocols, including six cycles of doxorubicin, bleomycin, vinblastine, and dacarbazine (ABVD); two cycles of gemcitabine, dexamethasone, and cisplatin (GDP); and two cycles of ifosfamide, carboplatin, and etoposide (ICE). Two relapses occurred after achieving complete metabolic response (CMR) in the first two protocols. The first relapse happened six months after ABVD completion, and the second occurred three months after GDP completion. 

 The last ICE protocol failed to control the disease, leading to progressive metabolic disease (PMD) and exhausting all available chemotherapy regimens. The patient disease then declared as refractory and the multidisciplinary clinic (MDC) decision was to start on intravenous pembrolizumab 200 milligram every three weeks. Prior to immunotherapy, ^18^F-FDG PET/CT was requested and revealed hypermetabolic lymphomatous disease affecting several supra- and infra-diaphragmatic lymph nodes (Figure 1b, arrows), left humeral shaft (Figure 1b, asterisk), and few splenic lesions (Figure 1b, double arrow). ^18^F-FDG PET/CT evaluation after five cycles resolved infradiaphragmatic lymph nodes and skeletal lesion, number regression in previous hypermetabolic splenic lesion with only single hypermetabolic focus remained visual at its lower border (Figure 1c, asterisk). Moreover, the previous hyper-metabolic mediastinal lymph node persisted (Figure 1c, curved arrow) while left axillary lymph node progressed (Figure 1c, arrow). Follow-up ^18^F-FDG PET/CT scan was advised to exclude pseudoprogression. Repeated ^18^F-FDG PET/CT scan after nine cycles revealed stable residual hypermetabolic focus within the spleen (Figure 1d, asterisk), as well as mediastinal lymph nodes (Figure 1d, curved arrow), with regressive features in the left axillary lymph node (Figure 1d, arrow), suggestive of prior pseudoprogression. The hypermetabolic left axillary lymph node progressed again on follow-up scan after cycle 13 (Figure 1e, arrow), despite stable splenic and mediastinal lesion (Figure 1e, asterisk and curved arrow), prompting the MDC to initiate targeted radiotherapy. The patient resumed pembrolizumab intake and received External Beam Radiation Therapy (EBRT) to the left axilla as (40 Grays/20 fractions). Follow up ^18^F-FDG PET/CT scan was suggestive of CMR (Figure 1f). Unfortunately, the patient developed recurrence within the spleen as well as in few lymph nodes on both sides of the diaphragm on the ^18^F-FDG PET/CT scan performed after cycles 22 (Figure 1g, annotations). The short-term follow-up ^18^F-FDG PET/CT scan after two more cycles showed resolution of all of the lesions except for stable hypermetabolic mediastinal lymph node (Figure 1h), suggestive of second episode of pseudoprogression at cycle 22. In ^18^F-FDG PET/CT scan performed after cycle 28, the patient developed progressive disease (Figure 1i, annotations), that stabilized on the follow up scans performed after cycle 31 and 35 respectively (Figure 1j, k; annotations). Notably, no dose escalation or de-escalation was done and the patient did not develop any side effects related to the ongoing immunotherapy.

## Discussion

 Approved in 2017 for HL treatment, pembrolizumab is an immune checkpoint inhibitor (ICI) targeting the programmed cell death receptor 1 (10). By blocking the interaction between programmed cell death receptor 1 and its ligand, programmed cell death ligand 1 (PD-L1), pembrolizumab restores antitumor immune responses, thereby potentially inhibiting tumor progression (10, 11). Clinical studies have demonstrated the efficacy of pembrolizumab against HL (10).

 The role of ^18^F-FDG PET/CT in evaluating responses to ICIs has been extensively investigated in recent years. The widely adopted Lugano classification has shown limitations in accurately assessing responses to ICI therapy, particularly in cases of pseudo-progression, necessitating the development of novel response assessment criteria (12). The Lymphoma Both LYRIC and RECIL incorporate both tumor size and metabolic activity to improve response assessment (5, 6). These criteria introduced new response patterns, including Indeterminate Response (IR) and Minor Response, which account for pseudoprogression (5, 6). While LYRIC and RECIL successfully characterized two pseudoprogression episodes in this case, neither provided definitive recommendations regarding disease recurrence following CMR. Adherence to these criteria would have led to unwarranted treatment discontinuation, given that subsequent imaging demonstrated disease stabilization (Figure 1 i-k).

 Nodal radiotherapy in combination with immunotherapy has shown promise as an effective treatment modality (13). In addition to targeting non-responding or residual lymph nodes, radiotherapy may enhance the efficacy of immunotherapy (13). The response observed in non-irradiated lymph nodes may be attributed to the abscopal effect (14). In this case, the patient received radiotherapy to the left axillary lymph node, which subsequently rendered CMR on follow-up imaging. The observed total CMR on the ^18^F-FDG PET/CT scan following cycle 13 (Figure 1f) may be attributed to both the synergistic effect of radiotherapy and the abscopal effect.

 Pseudoprogression, with an incidence of up to 10%, most frequently occurs within the initial 12 weeks of treatment (15). Notably, this patient experienced two distinct pseudoprogression episodes: the first following cycle 5 (Figure 1c, d) and the second after cycle 22, occurring more than one year after treatment initiation (Figure 1g, h). Immune-related adverse events (irAEs), which can affect multiple organ systems (e.g., thyroiditis, colitis, hepatitis, and arthritis), are generally not life-threatening and can be managed clinically (16). ^18^F-FDG PET/CT has proven valuable in the early detection of irAEs, facilitating timely diagnosis and intervention (3, 17). Notably, serial PET/CT scans in this patient 

did not reveal any evidence of irAEs, and the patient remained clinically stable throughout treatment.

 Despite disease relapse and subsequent progression following CMR while on ICI therapy, the disease stabilized on subsequent imaging (Figure 1 i-k). Furthermore, the current disease burden appears to be less extensive compared to baseline imaging prior to ICI initiation (Figure 1a-k)), with no evidence of new lesions. Given the presence of residual disease, treatment discontinuation was deemed inappropriate, and the most recent multi-disciplinary consensus recommended continuation of therapy with follow-up ^18^F-FDG PET/CT in three months.

 In conclusion, serial ^18^F-FDG PET/CT in evaluation of ongoing ICI represents an effective approach for guiding treatment decision. The identification of complex, heterogeneous response patterns during longitudinal assessment underscores the necessity for patient-tailored management strategies. Premature treatment discontinuation based on limited time point comparisons should be avoided, as serial imaging-integrating both baseline and interval scans-provides critical insights into tumor response dynamics. Further investigation in this field is highly welcomed.
